# Risk Factors for Failure of Direct Current Cardioversion in Patients with Type 2 Diabetes Mellitus and Atrial Fibrillation

**DOI:** 10.1155/2018/5936180

**Published:** 2018-03-12

**Authors:** Handrean Soran, Moulinath Banerjee, Jamal B. Mohamad, Safwaan Adam, Jan Hoong Ho, Shakawan M. Ismaeel, Shaishav Dhage, Akheel A. Syed, Ibrahem M. A. Abdulla, Naveed Younis, Rayaz A. Malik

**Affiliations:** ^1^Cardiovascular Trials Unit, The Old St Mary's Hospital, Hathersage Road, Manchester, UK; ^2^Cardiovascular Research Group, Core Technology Facility, University of Manchester, 3rd Floor, 46 Grafton Street, Manchester, UK; ^3^Bolton Diabetes and Endocrine Unit, Royal Bolton Hospital, Bolton, UK; ^4^Department of Medicine, School of Medicine, Duhok University, Duhok, Kurdistan Region, Iraq; ^5^Department of Medicine, Wirral University Teaching Hospital, Wirral, UK; ^6^Department of Endocrinology, Salford Royal Hospital, Salford, UK; ^7^Department of Medicine, Shar Hospital, Sulaymaniyah, Kurdistan Region, Iraq; ^8^Department of Diabetes and Endocrinology, University Hospital of South Manchester, Manchester, UK; ^9^Weill Cornell Medical College Qatar, Division of Medicine, Qatar Foundation, Education City, Doha, Qatar

## Abstract

**Introduction:**

Type 2 diabetes mellitus (T2DM) is a well-recognised risk factor for cardiovascular disease and the prevalence of atrial fibrillation (AF) is higher among patients with T2DM. Direct current cardioversion (DCCV) is an important management option in persistent AF. We sought to determine independent risk factors for immediate and short-term outcomes of DCCV for treatment of AF in patients with T2DM.

**Methods:**

Retrospective outcome analysis of DCCV for persistent AF in 102 T2DM patients compared with 102 controls.

**Results:**

DCCV was successful in 68 (66.6%) people with T2DM compared to 86 (84.3%) in the control group (*P* = 0.003). After initial successful cardioversion, only 38 (37.2%) T2DM patients remained in sinus rhythm compared to 63 (61.8%) in the control group (*P* = 0.007) at a median follow-up of 74.5 days (IQR 69.4–77.4). Multiple logistic regression analysis showed that the presence of T2DM (*P* = 0.014), digoxin use (*P* = 0.01), statin use (*P* = 0.005), left-atrial size (*P* = 0.01), and LV ejection fraction (*P* = 0.008) were independent risk factors for immediate DCCV failure. T2DM (*P* = 0.034) was an independent risk factor for AF relapse. Among patients with T2DM, previous DCCV (*P* = 0.033), digoxin use (*P* = 0.035), left-atrial size (*P* = 0.01), LV ejection fraction (*P* = 0.036), and HbA1c (*P* = 0.011) predicted immediate failure of DCCV whilst digoxin use (*P* = 0.026) was an independent risk factor for relapse of AF.

**Conclusion:**

T2DM, higher HbA1c, digoxin treatment, and structural and functional cardiac abnormalities are independent risk factors for immediate DCCV failure and AF relapse.

## 1. Introduction

Type 2 diabetes mellitus (T2DM) is a well-recognised risk factor for cardiovascular disease (CVD) [1]. The major manifestations of CVD are mainly macrovascular, but changes in the coronary microcirculation are well documented among patients with T2DM [2]. The prevalence of atrial fibrillation (AF), a common feature of CVD, is higher among patients with diabetes, when compared to patients without diabetes [3]. AF also contributes to increased morbidity and mortality in patients with T2DM [3, 4]. Though the debate between rate and rhythm control rages on [5], direct current cardioversion (DCCV) is still rooted within clinical guidelines as an important management option for AF especially in those with symptomatic and persistent AF [6]. There is an increasing recognition that patients who have diabetes and atrial fibrillation have a greater risk of ischaemic stroke and complications associated with anticoagulation. The success rates of both ablation and cardioversion are lower in those with diabetes [7]. In addition, modulation of the renin angiotensin system [8] and therapy with statins [9] may determine the risk of AF recurrence. We have previously reported that patients with T2DM are less responsive to DCCV; however, that cohort lacked robust control data [10]. We have therefore undertaken a case-control study to determine the independent risk factors for the immediate success rate and relapse following DCCV in patients with AF.

## 2. Patients and Methods

This was a retrospective case-control study in patients with persistent AF (pAF) attending the Cardiology Department for DCCV in a large district general hospital in Wirral, United Kingdom. We identified patients who had undergone DCCV between October 2001 and April 2007 (*n* = 624) using the hospital's electronic database. Relevant risk factor data were extracted from the medical records. The study was approved by the Wirral Research Ethical Committee.

*Inclusion Criteria. *All adult patients with documented pAF who underwent DCCV from October 2001 to April 2007 were included.

**Figure 1:**
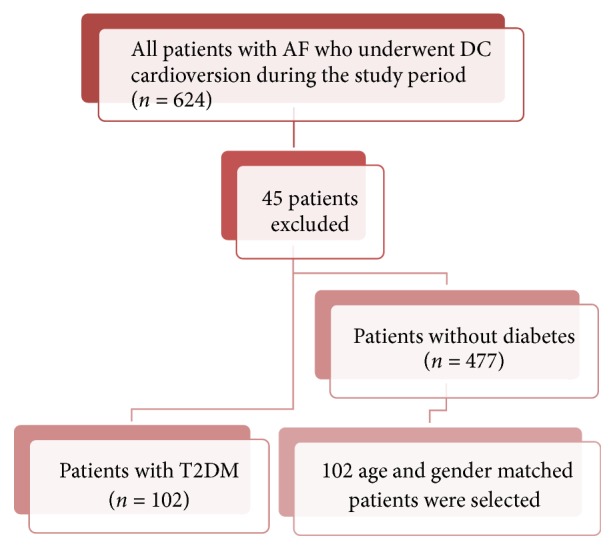
Study design and patient selection.

*Exclusion Criteria. *Patients with cardiac valvular disease (other than mild mitral and tricuspid valve regurgitation), rheumatic heart disease, previous heart valve surgery, congenital heart disease, patients with AF as a consequence of acute myocardial infarction (MI) or cardiac surgery, significant chronic kidney disease (defined as eGFR less than 60 ml/min/1.73 m^2^), AF secondary to thyrotoxicosis, patients who reverted to sinus rhythm (SR) before DCCV, and patients in whom relevant data were missing from the notes were excluded from the study. A total of 45 patients were excluded: 14 had valvular heart disease, two had history of heart valve replacement, three had AF after coronary artery bypass grafting, eight reverted to SR before attempted DCCV, two had AF secondary to thyrotoxicosis, five developed AF in the course of acute MI, and 13 were excluded because of missing data (Figure 1).

Of the 364 eligible patients, 102 had T2DM and of the 262 patients without T2DM 102 age and gender matched patients were selected as the control group for this study (Table 1).

All patients were anticoagulated for at least 6 weeks prior to cardioversion. International normalized ratio (INR) was checked at least weekly for the preceding 4 weeks prior to cardioversion and the dose of warfarin was adjusted to maintain the INR between 2.0 and 3.0 (target INR 2.5). Digoxin was stopped in all patients 48 hours before cardioversion and not recommenced in patients who had successful DCCV. Thyroid function tests, urea and electrolytes, full blood count, and cholesterol were checked in all patients. If amiodarone, sotalol, or flecainide were prescribed, patients continued on these until reviewed in clinic. All patients were reviewed 3 to 4 hours after DCCV (prior to discharge) and subsequently in the outpatient clinic. Clinical examination and a 12-lead electrocardiogram (ECG) were performed at each review.

Successful DCCV was defined by an ECG 3 hours after DCCV showing sinus rhythm. Persistent AF was defined as episodes that failed to self-terminate spontaneously and lasted for longer than 7 days but could be converted to SR with pharmacological or electrical cardioversion. The duration of AF was the period (in weeks) from the first day of diagnosis of the index atrial fibrillation supported by ECG to the day of DCCV. In patients who had a successful cardioversion, the arrhythmia status on the day of first outpatient follow-up was documented to assess differences between the two groups. All patients with a documented history of hypertension or three recent blood pressure readings greater than 160/90 mmHg were regarded as having hypertension. All patients on lipid-lowering treatment or with a total serum cholesterol measurement greater than 5.2 mmol/l were regarded as having hyperlipidaemia. In patients with diabetes, a mean HbA1c of the three most recent measurements was used. HbA1c values were measured by affinity chromatography using a commercial kit (BioRad, UK; nondiabetic range 22–40 mmol/mol [4.2–5.8%]). Echocardiograms were performed in the Department of Cardiology, Wirral University Hospitals NHS Foundation Trust (using Philips-Sonos 5500 and Philips-Sonos 5400). All echocardiogram operators had British Society of Echocardiography accreditation. An estimate of left ventricular ejection fraction (LVEF) was made using automated software. Left ventricular hypertrophy (LVH) was diagnosed using echocardiography by measuring the thickness of the intraventricular septum and posterior wall. Using an anteroapical position, monophasic DCCV was performed on an elective basis with a Hewlett Packard device. One hundred joules was used as the initial energy current and titrated according to response to a maximum of 360 J.

Statistical analysis SPSS v22.0 was used to analyse the data. Data were expressed as frequency and percentage for categorical data and mean ± SD for continuous data. We used recurrence of AF at the first outpatient follow-up as the dependent outcome. Univariate model with a cut-off of *P* < 0.05 was used to identify potentially significant factors. Multiple regression analyses using the factors identified above were used to identify independent risk factors for recurrence of AF following successful DC cardioversion. A *P* value < 0.05 was statistically significant.

## 3. Results

102 patients with T2DM with a mean diabetes duration of 5.5 (95% CI: 4.6–6.4) years and 102 age and gender matched control patients without T2DM underwent DCCV for nonvalvular AF (Table 1). Apart from the duration of AF prior to DCCV and use of renin angiotensin system (RAS) inhibitors, there were no significant differences between the two groups.

**Figure 2:**
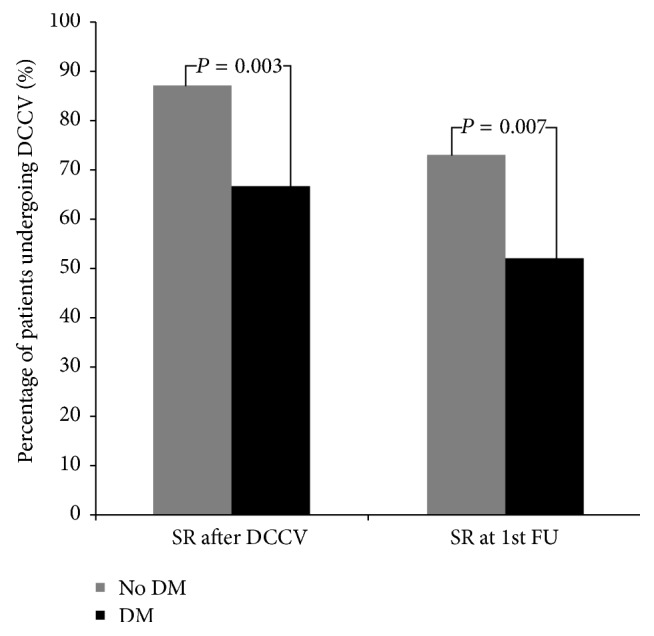
The percentage of patients with and without T2DM in sinus rhythm immediately after DC cardioversion and at the first follow-up visit. There were a higher percentage of patients achieving sinus rhythm immediately after DC cardioversion without diabetes than with T2DM, (*P* = 0.003) and at the first follow-up visit (*P* = 0.007).

DCCV resulted in immediate (prehospital discharge) success in 75.5% of all patients. Of the 102 patients with T2DM, 68 (66.6%) achieved immediate cardioversion to sinus rhythm compared to 86 (84.3%) of 102 patients without T2DM (OR: 0.372, 95% CI: 0.19–0.73, and *P* = 0.003). The median time to the 1st follow-up visit was 75 [95% CI: 74.0–76.0] days; of the 68 patients with T2DM who had achieved immediately successful DCCV, 38 (55.9%) had maintained sinus rhythm compared to 63 of 86 (73.3%) patients without T2DM [OR: 0.398, 95% CI: 0.203–0.730, and *P* = 0.007] (Figure 2).

### 3.1. Multiple Regression Analysis

After adjusting for age, gender, duration of AF, previous DCCV, smoking history, preexisting atherosclerotic disease, dyslipidaemia, alcohol excess, RAS inhibitor usage, any heart-rate limiting agent usage, and presence of LVH, T2DM (*β*: −0.144, 95% CI of *β*: −0.259 to −0.029, *P* = 0.014), use of digoxin (*β*: −0.162, 95% CI of *β*: −0.285 to −0.040, and *P* = 0.01), statin use (*β*: −0.204, 95% CI of *β*: −0.347 to −0.062, and *P* = 0.005), left-atrial size (*β*: −0.103, 95% CI of *β*: −0.181 to −0.025, and *P* = 0.01), and LVEF (*β*: 0.006, 95% CI of *β*: 0.002 to 0.011, and *P* = 0.008) independently influenced attainment of SR immediately after DCCV (Table 2). A further model which also included the time between DCCV and the first follow-up visit as well as the aforementioned factors found T2DM (*β*: −0.186, 95% CI of *β*: −0.357 to −0.014, and *P* = 0.034) to independently and inversely influence the persistence of SR at the 1st visit following DCCV.

In patients with T2DM, after adjusting for age, gender, duration of AF, smoking history, preexisting atherosclerotic disease, obstructive airways disease, dyslipidaemia, alcohol excess, use of statins, RAS inhibitors, and rate limiting agents, presence of left ventricular hypertrophy and microvascular disease (as defined by presence of any degree of retinopathy or neuropathy documented in the medical notes as part of annual screening and assessment, nephropathy defined as documented microalbuminuria with urinary albumin: creatinine ratio of >3 mg/mmol on two occasions), previous DCCV (*β*: −0.558, 95% CI of *β*: −1.071 to −0.46, and *P* = 0.033), use of digoxin (*β*: −0.206, 95% CI of *β*: −0.397 to −0.015, and *P* = 0.035), left-atrial size (*β*: −0.184, 95% CI of *β*: −0.324 to −0.045, and *P* = 0.01), LV ejection fraction (*β*: 0.007, 95% CI of *β*: 0.001 to 0.014, and *P* = 0.036), and HbA1c (*β*: −0.104, 95% CI of *β*: −0.183 to −0.025, and *P* = 0.011) independently influenced attainment of sinus rhythm immediately following DCCV (Table 3). A further model which included the time between DCCV and the first follow-up visit along with the aforementioned factors found digoxin use (*β*: −0.413, 95% CI of *β*: −0.774 to −0.051, and *P* = 0.026) to independently and inversely influence persistence of sinus rhythm at the 1st visit following DCCV.

## 4. Discussion

Conceptually, consequences of AF can be mostly ameliorated by attainment of sinus rhythm, though this has been debated [11]. When chemical cardioversion fails electrical cardioversion may be attempted depending on physician and patient preference [6]. In the present study the overall immediate prehospital discharge success rate of DCCV in our patients was 75.5%, which is comparable to previous reports [12]. Possible reasons for failure to attain and maintain sinus rhythm have been discussed by Frick et al. [12], but their list does not include diabetes. Indeed, there are very limited data regarding success rate of DC cardioversion in patients with diabetes. Thus in a small cohort of 48 patients, where the efficacy of atorvastatin in preventing the recurrence of AF was investigated, diabetes was found to be a significant risk factor influencing the risk of recurrence of AF [13].

We demonstrate, for the first time, a lower success rate of both immediate cardioversion and subsequent maintenance of sinus rhythm in patients with T2DM. Indeed, further regression analysis confirmed that the diagnoses of T2DM and HbA1c among the patients with T2DM are independent risk factors for DC cardioversion failure. Poorer glycaemic control is a known risk factor for CVD [1] and the risk of developing atrial fibrillation [14]. Furthermore, patients with diabetes and AF are more likely to develop cardioembolic stroke [15], as well as having increased morbidity and mortality [16]. It is therefore postulated that limiting the onset of AF and reversion to sinus rhythm may result in improved outcomes [14], although more conclusive evidence for this is required. Improved glycaemic control per se was not shown to impact on the incidence of atrial fibrillation in the recent ACCORD study [16]. A recent study has shown that a higher HbA1c is associated with an increased risk of recurrence of atrial tachyarrhythmia in patients with T2DM undergoing catheter ablation [17]. However, the impact of T2DM and especially the impact of hyperglycaemia on the success of DCCV have not been described to date.

The presence of other microvascular and macrovascular complications may directly influence the development and chronicity of atrial fibrillation in people with diabetes [18]. Indeed, the prevalence of AF was shown to be greater in patients with diabetic autonomic neuropathy compared with patients who have diabetes but without neuropathy [19]. Importantly, cardiac autonomic neuropathy can influence the onset of AF as well as prognosis following DCCV [20]. After adjustment for preexisting macrovascular and microvascular disease, HbA1c remained an independent adverse risk factor for the success of DCCV. Recurrent AF has a low success rate of cardioversion which is secondary to atrial remodeling during AF [21]. This in itself was found to be an independent risk factor for unsuccessful treatment of AF in patients with T2DM.

It is well known that patients treated with digoxin have a lower chance of spontaneous reversions to sinus rhythm, as well as successful DCCV [22]. In this study we show a similar result, particularly in patients with T2DM. In this group it was found to be an independent risk factor influencing the immediate success as well as maintenance of sinus rhythm at follow-up. Why digoxin use may influence more adverse outcomes is not clear, but altered myocardial calcium homeostasis in patients with T2DM may be a possible explanation [23].

Statins have also been shown to have a favourable impact on attaining and maintaining sinus rhythm in patients with AF [13]. However, we found that concomitant statin usage had a favourable impact on the whole group, but not among the patients with T2DM. This may be due to the myocardial electrophysiological effect of statins which could be mediated by a reduction in CRP [24], interleukins [25], catecholamines [26], and altered cell membrane properties [27], which may well be further attenuated in patients with T2DM.

Structural cardiac alteration with increased LA size [28] in particular is known to influence success in maintaining sinus rhythm in patients undergoing DC cardioversion for AF. Thus AF per se is thought to lead to left-atrial remodeling [28]. We confirm this association in patients with and without T2DM. The other major haemodynamic consequence of persistent AF is its impact on LV systolic function, which can improve after reversion to sinus rhythm [29]. The main mechanism for such a reduction may be the tachycardia-induced LV systolic dysfunction, due to reduced myocardial calcium secondary to shortened diastole [30].

The present study is the largest case-control study identifying a range of risk factors, determining immediate and longer-term outcomes following DC cardioversion in patients with T2DM. A limitation of this study is that the technique for cardioversion used was monophasic DCCV whilst current clinical guidelines advocate the use of biphasic DCCV due to superior efficacy and fewer energy requirements [6]. The success rate for DCCV at the first visit in our study as aforementioned was similar to previous reports [12] and interestingly for patients without T2DM the proportion of those who attained sinus rhythm was 84% which is very similar to the results in a previous relatively large study in which the efficacy of monophasic DCCV was found to be inferior to biphasic DCCV (84% versus 95% success rate) [31]. In that study, out of the 229 patients who had monophasic DCCV for treatment of AF, only 9 had a history of diabetes [31]. Another limitation of this study was that the duration of AF precardioversion was significantly longer in the group of patients with T2DM compared to those without T2DM. Duration of AF is known to be a risk factor for inability to achieve sinus rhythm on first attempt as well as recurrence of AF at follow-up using DCCV treatment [12]. Duration of AF was therefore adjusted in the regression model when determining independent risk factors for both immediate success and relapse. Ventricular rate prior to DCCV was not assessed as a significant proportion of patients were on rate limiting medications. The prevalence of rate limiting medication use, however, did not differ between groups with and without T2DM. We acknowledge that due to the retrospective design of this study certain relevant information such as body mass index was not readily available to us and would have added to the data interpretation. Despite consideration of all of these factors, we believe this study provides important insights into the potential basis for poorer outcomes in patients with T2DM undergoing DCCV.

## 5. Conclusion

The presence of T2DM and the degree of hyperglycaemia as represented by HbA1c are independent risk factors for immediate and medium-term failure of DCCV in patients with AF.

## Figures and Tables

**Table 1:** Baseline characteristics of patients. Data are mean (SD). AF, atrial fibrillation; DC, direct current; CAD, coronary artery disease; PAD, peripheral arterial disease; ACE, angiotensin converting enzyme; ARB, angiotensin receptor blocker; LA, left atrium; LV, left ventricle.

Characteristics	Patients without T2DM (*n* = 102)	Patients with T2DM (*n* = 102)	*P*
Age, years	69.4 ± 7.8	68.9 ± 7.6	NS
Gender ratio, M : F	0.73	0.73	NS
Duration of AF prior to DC cardioversion, weeks	14.7 ± 8.8	20.3 ± 18.8	0.007
Previous DC cardioversion, *n* (%)	6 (5.9)	3 (2.9)	NS
History of smoking, *n* (%)	2 (2.0)	8 (7.8)	0.052
Preexisting atherosclerotic disease (CAD, stroke, and PAD), *n* (%)	45 (44.1)	50 (49.0)	NS
Obstructive airways diseases, *n* (%)	11 (10.8)	8 (7.8)	NS
Hyperlipidaemia, *n* (%)	36 (35.3)	42 (41.2)	NS
History of alcohol excess, *n* (%)	4 (3.9)	2 (2.0)	NS
ACE-inhibitor or ARB use, *n* (%)	40 (39.2)	63 (61.8)	0.001
Amiodarone use, *n* (%)	46 (45.1)	40 (39.2)	NS
Flecainide use, *n* (%)	1 (1.0)	2 (2.0)	NS
Sotalol use, *n* (%)	4 (3.9)	4 (3.9)	NS
Beta-blocker use, *n* (%)	33 (32.4)	27 (26.5)	NS
Calcium channel blocker use, *n* (%)	23 (22.5)	33 (32.4)	NS
Digoxin use, *n* (%)	29 (28.4)	36 (35.3)	NS
Statin use, *n* (%)	29 (28.4)	32 (31.4)	NS
LA size, cm	4.3 ± 0.8	4.3 ± 0.7	NS
LV ejection fraction, %	56.2 ± 11.8	54.0 ± 12.4	NS
Presence of LV hypertrophy, %	14 (13.7)	10 (9.8)	NS
1st follow-up visit after DC cardioversion, days	75.2 ± 6.9	74.9 ± 7.5	NS

**Table 2:** Regression model assessing factors affecting attainment of sinus rhythm immediately after DCCV. DCCV, direct current cardioversion; LA, left atrium; LVEF, left ventricular ejection fraction; T2DM, type 2 diabetes mellitus.

Variables	*β*-Coefficient	*P*
T2DM	−0.144	0.014
Digoxin use	−0.162	0.010
Statin use	−0.204	0.005
LA size	−0.103	0.010
LVEF	0.006	0.008

**Table 3:** Regression model assessing factors affecting attainment of sinus rhythm immediately after DCCV in patients with T2DM. DCCV, direct current cardioversion; HbA1c, glycated haemoglobin; LA, left atrium; LVEF, left ventricular ejection fraction; T2DM, type 2 diabetes mellitus.

Variables	*β*-Coefficient	*P*
Previous DCCV	−0.558	0.033
Digoxin use	−0.206	0.035
LA size	−0.184	0.010
LVEF	0.007	0.036
HbA1c	−0.104	0.011

## Additional Points

*Key Messages*. (i) This study shows that type 2 diabetes negatively impacts on the success of direct current cardioversion (DCCV) for atrial fibrillation (AF). (ii) The presence of T2DM is an independent risk factor for the relapse of AF after treatment. (iii) We report that in patients with type 2 diabetes glycated haemoglobin is a predictor for immediate DCCV failure in AF.
